# Congenital second branchial cleft anomalies in children: A report of 52 surgical cases, with emphasis on characteristic CT findings

**DOI:** 10.3389/fped.2023.1088234

**Published:** 2023-03-03

**Authors:** Wei Chen, Yilong Zhou, Mengrou Xu, Rong Xu, Qingyu Wang, Hongming Xu, Jiarui Chen, Xiaoyan Li

**Affiliations:** ^1^ Department of Otolaryngology-Head and Neck Surgery, Shanghai Children’s Hospital, School of Medicine, Shanghai Jiao Tong University, Shanghai, China; ^2^ Department of Pediatric Otorhinolaryngology, Shenzhen Hospital, Southern Medical University, Shenzhen, China; ^3^ Department of Radiology, Shanghai Children’s Hospital, School of Medicine, Shanghai Jiao Tong University, Shanghai, China; ^4^ Department of Pathology, Shanghai Children’s Hospital, School of Medicine, Shanghai Jiao Tong University, Shanghai, China

**Keywords:** congenital second branchial cleft anomalies, Bailey classification, sternocleidomastoid muscle, submandibular gland, carotid sheath

## Abstract

**Objective:**

The objectives of this study was to review the clinical features and surgical treatment outcomes of congenital second branchial cleft anomalies (CSBCAs) and to investigate the characteristic computed tomography (CT) findings of CSBCAs.

**Methods:**

We conducted a retrospective study of 52 children who were referred to Shanghai Children's Hospital from October 2014 to December 2021 diagnosed as CSBCAs.

**Results:**

There were 36 males and 16 females. Of them, 35 patients were presented as having a skin pit at birth or discharge from the skin opening on the lateral neck, and 17 patients presented with an asymptomatic or painful mass. The typical CT features of CSBCAs included isolated and homogeneously hypodense cystic lesions surrounded by a uniformly thin, smooth wall. CSBCAs were generally located at the anteromedial border of the sternocleidomastoid muscle, posterior to the submandibular gland, and lateral to the carotid sheath. All patients were treated surgically and only one case underwent ipsilateral tonsillectomy. After a median follow-up of 30 (range 4–90) months, no recurrence or complications were observed.

**Conclusions:**

The CSBCAs show some characteristic CT findings, which can help clinicians diagnose and plan surgical strategies. High ligation of the lesions is sufficient for complete excision of CSBCAs.

## 1. Introduction

Incomplete obliteration of branchial clefts and pouches during embryogenesis leads to branchial cleft anomalies (BCAs), which is the most widely accepted theory (1, 2). BCAs are second only to thyroglossal duct cysts (TGDCs) as the most common congenital cervical masses (3, 4). Because first, third, and fourth BCAs are rare, congenital second branchial cleft anomalies (CSBCAs) make up 85%–95% of all BCAs (1, 2, 5). CSBCAs may present as fistulas, cysts, or sinuses, and they usually present with nonspecific symptoms, including discharge from the skin opening, neck swelling, or recurrent infections (6–8). The lesions of CSBCAs may vary significantly from being short extending just up to the surface of sternocleidomastoid muscle (SCM), or up to carotid sheath, to a longer tract extending through carotid bifurcation up to pharyngeal constrictor muscles, or to the palatine tonsil (3, 8). The definitive treatment of CSBCAs is surgical excision (2). Success primarily depends on accurate definition and meticulous surgical resection of the tract, which can be sometimes challenging, particularly in multiply infected cases (9). Clinically, the CSBCAs are often misdiagnosed and need to be differentiated from TGDC, congenital first branchial cleft anomaly (CFBCAs), congenital pyriform sinus fistula (CPSF), lymphatic malformations (LMs), and other cystic neck lesions (1, 8). The above diseases have certain characteristic clinical and imaging appearances, and familiarity which can assist in clinical diagnosis (2, 10, 11).

Correct diagnosis and complete resection depend on the understanding of anatomy (1), which is extremely important to avoid inappropriate surgery and multiple procedures (12, 13). Herein, we investigated the clinical characteristics, the typical computed tomography (CT) findings, and surgical treatment outcomes of children with CSBCAs, so as to realize rapid clinical diagnosis and formulate surgical plan.

## 2. Materials and methods

### 2.1. General information

We conducted a retrospective analysis of pediatric patients with CSBCAs who underwent surgery at the Department of Otolaryngology-Head and Neck Surgery from October 2014 to December 2021. A summary of the 52 patients [36 males (69.2%) and 16 females (30.8%); age at presentation range, 1 day–13 years, median, 1 day; ages at operation range, 7 months–14 years, mean, 44 ± 16 months] is shown in Table 1. Collected data included patient demographics such as sex, symptoms, infections, treatment history, age at presentation, age at operation, location and size, preoperative CT, operative details, histopathological diagnosis, postoperative complications, length of follow-up, and recurrence. This study was approved by our institutional Research Ethics Board (Approval Letter of Ethics Review Committee, Children's Hospital of Shanghai/Shanghai Children's Hospital, Shanghai Jiao Tong University; Approval No: 2022R069-E01.Validity of the approval: 2022-06-08—2023-06-07]. The requirement for informed consent was waived due to the retrospective nature of this study.

**Table 1:** Clinical summary of our patients with CSBCAs.

CSBCAs	*N*
Gender (male/female)	36/16
Location (left/right/bilateral)	15/26/11
Type (cyst/sinus/fistula)	17/32/3
**Clinical manifestations**	
Painless lateral neck swelling	8
Lateral neck swelling with pain	9
Discharge from the skin opening	35
Infection history	57.7% (30/52)
History of incision and drainage/aspiration	19/1
Surgical history	1
Age at presentation (months)	Median: 0.03; Range: 0.03–156
Age at operation (months)	Mean: 44 ± 16; Range: 7–168
Contrast-enhanced CT	52
Diagnostic accuracy	86.5% (45/53)
US	37
Diagnostic accuracy	10.8% (4/37)
Size (cm)	Mean: 2.3 ± 1.4; ranged: 0.4–6.1
Bailey classification (type I/II/III/IV)	10/35/4/3
Ipsilateral tonsillectomy	1
**Histopathological structure**	
Squamous epithelium	20
Ciliated columnar epithelium	13
Mixed	19
Follow-up (months)	Median: 30; range: 4–90
Complications	NO
Recurrence	NO

CSBCAs, congenital second branchial cleft anomalies; CT, computed tomography; US, ultrasonography.

### 2.2. Enhanced CT

The CT scan (GE LightSpeed VCT, United States) was performed on the neck, with a layer thickness of 0.625 mm, interval of 2.5 mm, pitch of 0.984, tube voltage of 100 kV, and tube current of 240 mA. Contrast enhancement was performed *via* iohexol administration, and contrast-enhanced CT images were obtained from all 52 patients. Coronal and transverse multiplanar reconstruction CT images were reconstructed with 3-mm section thickness. Uncooperative cases were given 10% chloral hydrate (0.5 ml/kg) orally prior to the examinations (10). We mainly focused on the adjacent relationship between the lesion and SCM, submandibular gland (SMG), and carotid sheath.

### 2.3. Bailey classification

**Figure 1:**
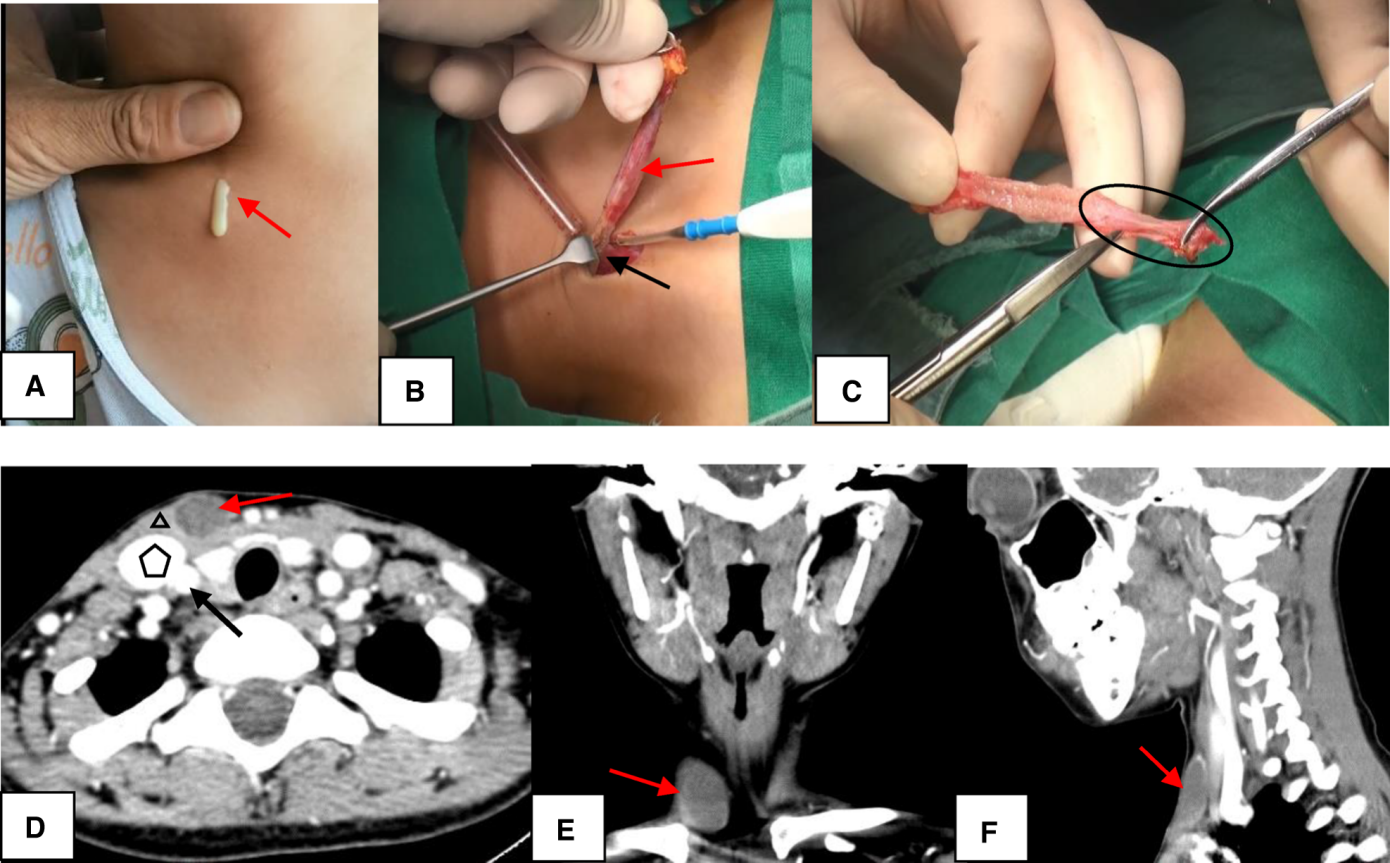
The Bailey type I. (**A**) The sinus was presented as having a skin pit on the lateral neck and discharge from the skin opening (red arrow). (**B**) The sinus (red arrow) terminated at the surface of SCM (black arrow). (**C**) The end of the sinus closed to form a muscle bundle (circle) ending at the surface of SCM. (**D–F**) On CT, the lesion (red arrow) lied along anterior surface of SCM (triangle) just deep to platysma, not in contact with the carotid sheath (pentagon: internal jugular vein; black arrow: arteria carotis communis). D, axial position; E, coronal position; F, sagittal position; SCM, sternocleidomastoid muscle; CT, computed tomography.

**Figure 2:**
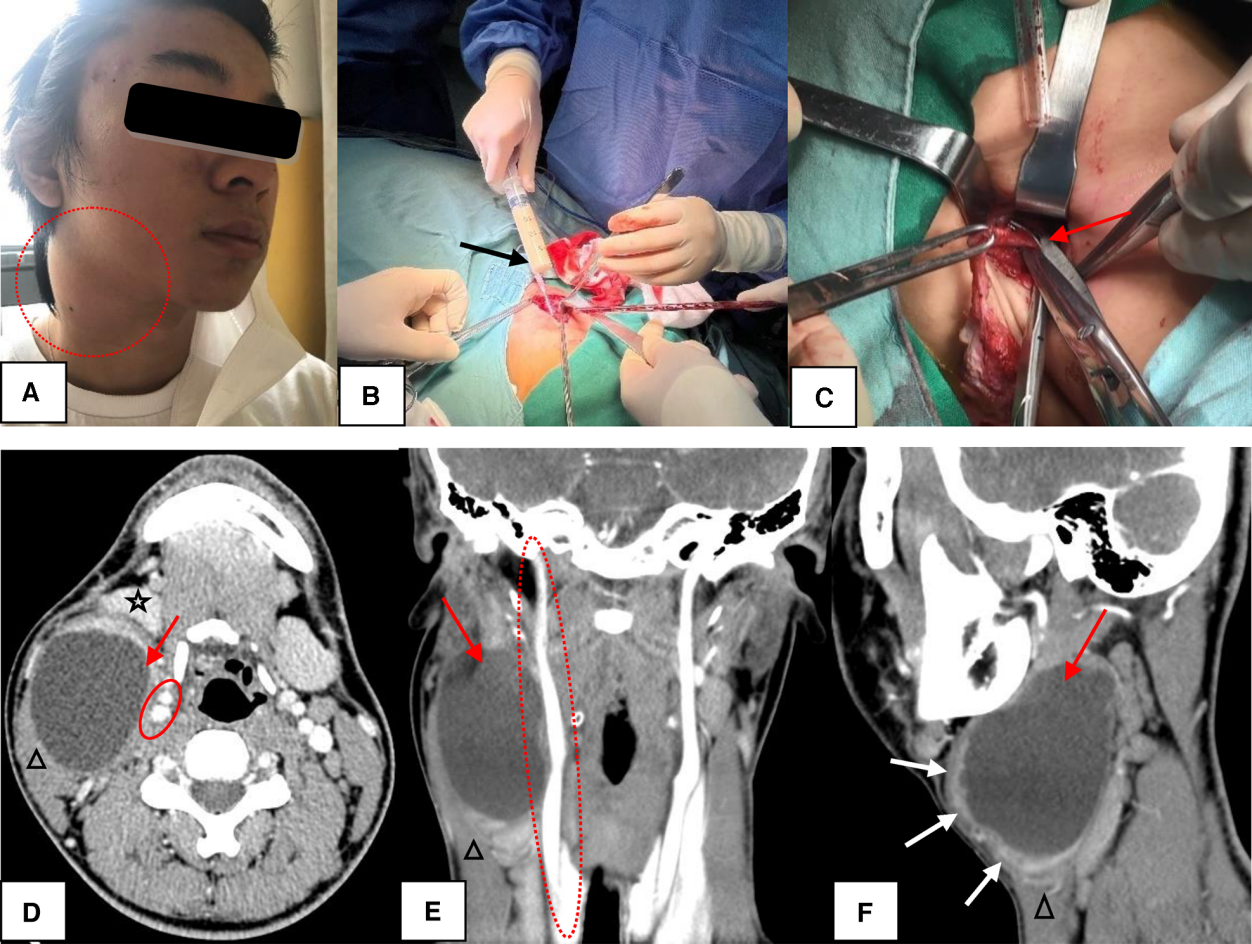
The Bailey type II. (**A**) The lesion presented as a painful mass in the right neck (circle). (**B**) During the operation, the mass contained a large amount of rice soup like liquid (black arrow). (**C**) High ligation of the sinus (red arrow) was sufficient for complete excision of CSBCAs. (**D–F**) CT scans showed a well-circumscribed and homogeneously low-density cystic mass (red arrow) with uniformly thin, smooth wall. If infected, the cyst wall became thickened and irregular (F, white arrow). The lesion was found in the “classic” location: along the anteromedial border of the SCM (triangle), lateral to the carotid sheath (circle), and posterior to the SMG (five-pointed star). D, axial position, E, coronal position, F, sagittal position; CSBCAs, congenital second branchial cleft anomalies; SCM, sternocleidomastoid muscle; CT, computed tomography.

**Figure 3:**
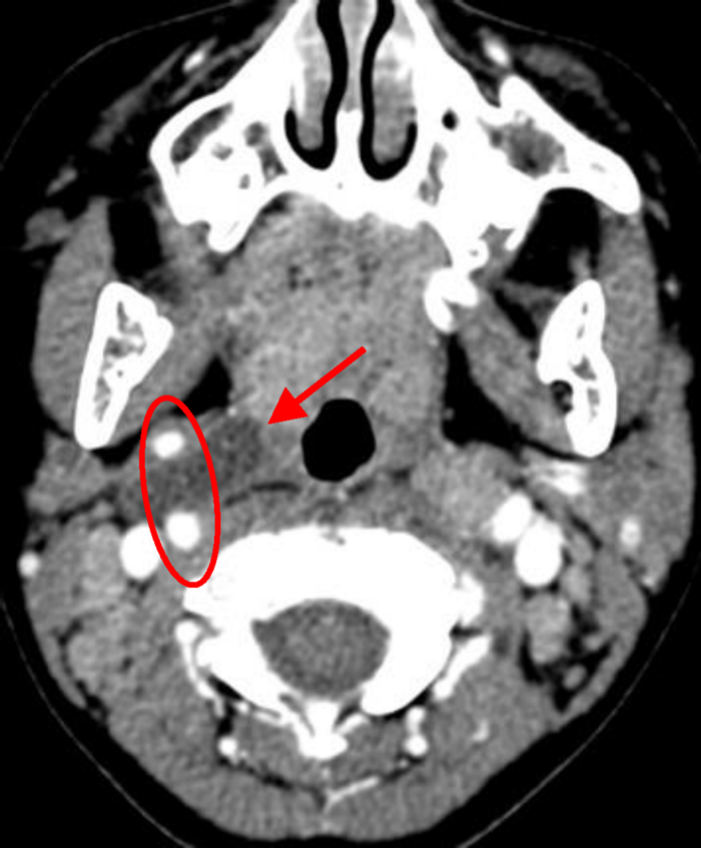
The Bailey type III. The lesion (red arrow) extended medially between the bifurcation of internal and external carotid arteries (circle) to the lateral pharyngeal wall.

**Figure 4:**
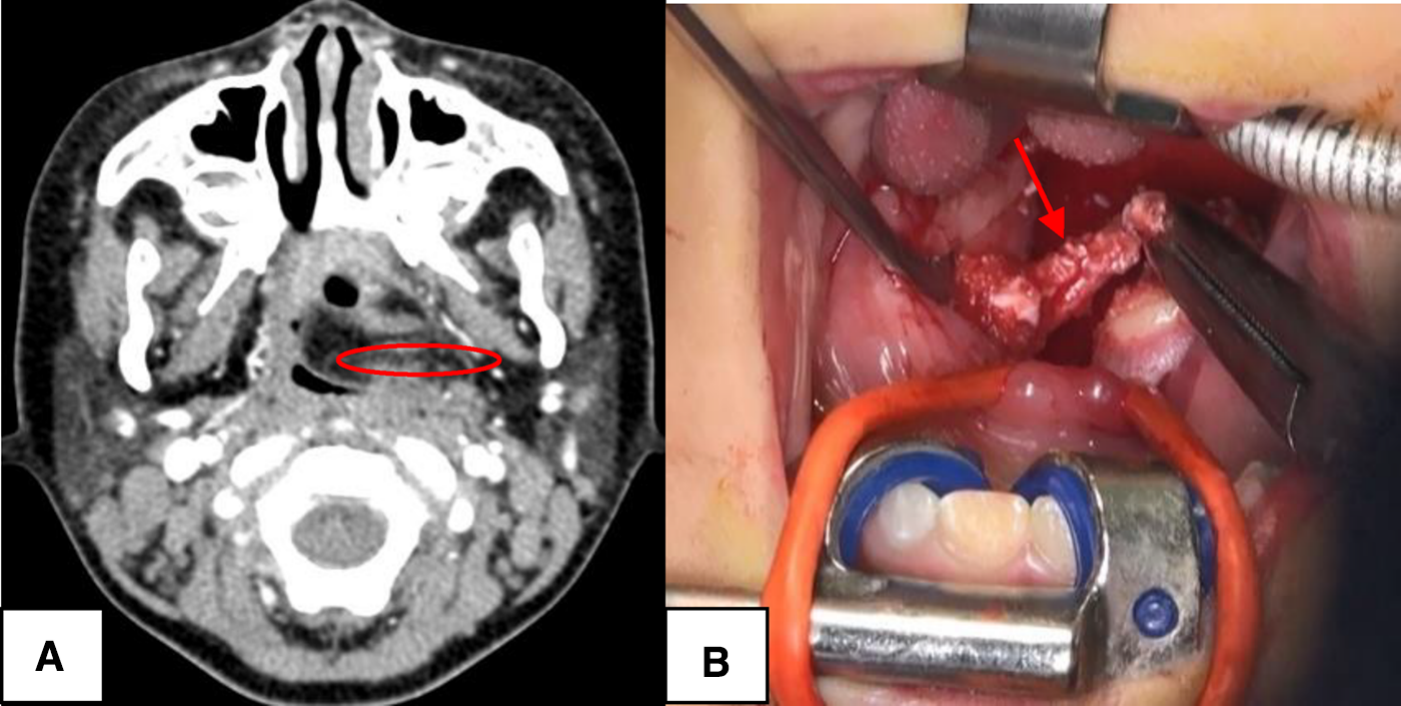
The Bailey type IV. (**A**) The lesion (red circle) was toward ipsilateral pharynx to end up to the palatine tonsil, blocking the oropharynx. (**B**) The case underwent complete surgical excision with ipsilateral tonsillectomy (red arrow), *via* external cervical approach combined with oral approach.

CSBCAs were classified into the following four subtypes on the basis of the Bailey classification: (i) the most superficial subtype, which reached as deep as the platysma surface and lies along the anterior surface of the SCM, but not in contact with the carotid sheath (Figure 1); (ii) the most common subtype, identified anterior to the SCM, posterior to the SMG, and lateral to the carotid sheath (Figure 2); (iii) extended medially between the bifurcation of the external and internal carotid arteries, lateral to the pharyngeal wall (Figure 3); and (iv) arose in the pharyngeal mucosal space and opened into the pharynx (1, 3, 7, 8, 14) (Figure 4).

### 2.4. Surgical operation

During the noninfectious stage, the surgery was carried out under general anesthesia with endotracheal intubation, with access through lateral neck incision. For Bailey type I, the lesion terminated on the surface of SCM and was completely removed (Figure 1B). High ligation of the lesion was sufficient for complete excision of CSBCAs as Bailey type II (Figure 2C). The carotid sheath should be protected during operation. For Bailey types III and IV, if the lesion did not block the oropharynx, we just performed high ligation (2, 9, 15). Only one patient with oropharyngeal obstruction underwent surgical resection combined with ipsilateral tonsillectomy (Figure 4B). The postoperative specimens were carried to pathological examination. Pressure bandage and negative pressure drainage tube were placed after surgery for 48–72 h. All patients received standard guidance of postoperative antibiotics, usually amoxicillin 40 mg/kg/dose twice daily, lasting about 7 days.

### 2.5. Follow-up

Patients were seen 1 month postoperatively by an otolaryngologist for clinical examination. The neck was observed for redness and swelling, pharyngeal fistula, hoarseness, cough, and recurrence. Long-term assessment of complications and recurrence were collected by chart review.

### 2.6. Statistical analysis

The collected data were analyzed by SAS 9.13 software package. The measurement data of normal distribution was expressed as mean ± standard deviation $\left(\bar{\chi }\;\pm \;s\right)$, and that of skew distribution was expressed as median. A *χ*^2^ test of R × C contingency table was used to analyze whether there were differences in age, type, gender, and location among the four Bailey types. *P* < 0.05 was considered statistically significant.

## 3. Results

The CSBCAs were found on the right side in 26 patients (50.0%), on the left side in 15 patients (28.8%), and bilaterally in 11 patients (21.2%). Of the 52 patients, CSBCAs presented as fistula (3/52), sinus (32/52), and cysts (17/52). Thirty-five patients (67.3%) with a branchial cleft fistula/sinus presented as having a skin pit on the lateral neck at birth or discharging from the skin opening (including 21 cases with infection and 14 cases infection-free), and 17 patients (32.7%) with a branchial cleft cyst showed an asymptomatic mass (8/17) or recurrent infections (9/17) in the lateral neck region. Thirty patients (57.7%) had a history of infection, including 19 patients who underwent 1–3 times incision and drainage, 1 case of aspiration, and 1 case of surgical resection at a local hospital. The remaining 22 cases (42.3%) had no history of infection, including 14 fistulas/sinuses and 8 cysts. Fourteen children (27.0%) had a clinically relevant misdiagnosis preoperatively, including four LMs, four neck abscess, two TGDC, two CPSF, one bronchogenic cyst, and one ectopic thymus.

After statistical analysis, Bailey classification had nothing to do with gender, location, and age (*P* > 0.05). However, Bailey type I was mostly cyst type, while Bailey types II, III, and IV were fistula/sinus type, with statistical difference (*P* < 0.05) (Table 2).

**Table 2:** Statistical analysis for gender, location, type, age, and Bailey classification.

Bailey classification	I	II	III	IV	*P*
**Gender**					
Male	8	23	3	2	0.8486
Female	2	12	1	1	
**Location**					
Left	2	12	0	1	0.0621
Right	8	16	1	1	
Bilateral	0	7	3	1	
**Type**					
Cyst	6	11	0	0	0.0040
Sinus or fistula	4	24	4	3	
**Age (year)**					
0–3	8	29	4	3	0.6845
>3	2	6	0	0	

Fifty-two patients routinely underwent preoperative contrast-enhanced CT to determine the extent of the lesion and rule out other diseases. Forty-five patients (86.5%) were correctly diagnosed preoperatively by enhanced CT. The size of CSBCAs ranged from 0.4 to 6.1 cm (mean, 2.3 cm ± 1.4 cm). Typical CT findings included well-circumscribed and homogeneously hypodense cystic masses surrounded by a uniformly thin, smooth wall. Conversely, for infected CSBCAs, the cyst wall became thickened and irregular (Figure 2F). CSBCAs were generally located at the anteromedial border of the SCM, posterior to the SMG, and lateral to the carotid sheath in our series. Thirty-seven patients underwent neck ultrasonography (US). US showed 22 cases of benign cystic mass and 7 cases of fistula, and only 4 cases were accurately diagnosed, so the diagnostic accuracy (10.8%, 4/37) was very low.

**Figure 5:**
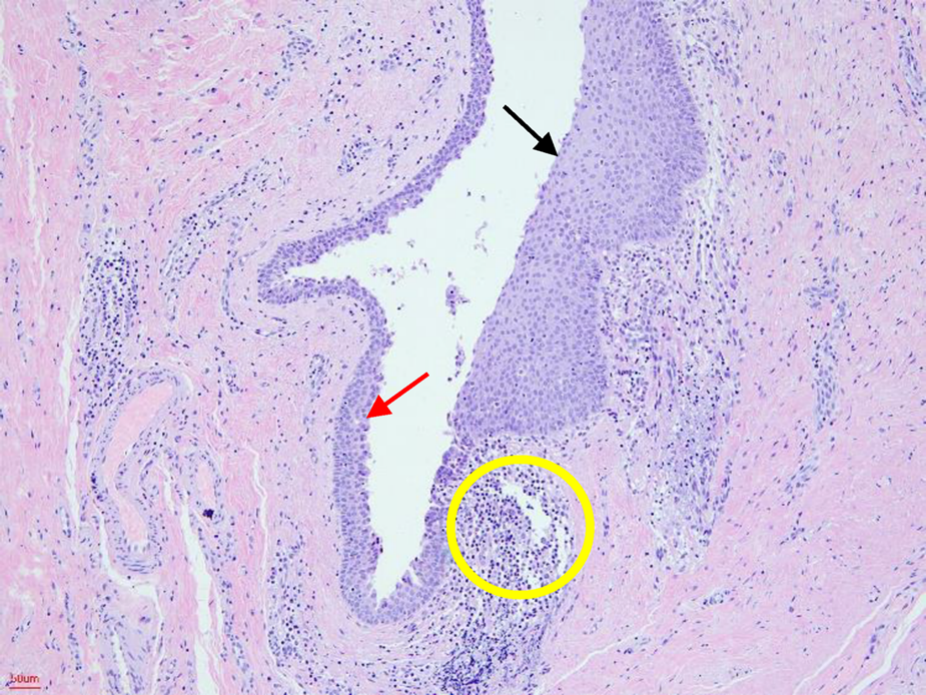
Histopathological examination. The cystic wall was lined by stratified squamous epithelium (black arrow) and columnar respiratory epithelium (red arrow). Lymphocyte aggregation can also be seen (yellow circle) (HE, hematoxylin-eosin staining; 100×).

All 52 cases underwent surgical excision, including 1 case with ipsilateral tonsillectomy. Histopathologically (Figure 5), 20 cases (38.5%) are lined by stratified squamous epithelium, 13 cases (25%) by columnar respiratory epithelium, and 19 cases (36.5%) were mixed. Bailey subtypes I, II, III and IV were present in 10 (19.2%), 35 (67.3%), 4 (7.7%), and 3 (5.8%) case, respectively. No recurrence or complications were observed after a median follow-up of 30 (range 4–90) months (Table 1).

## 4. Discussion

BCAs are the second most common pediatric head and neck congenital lesions, accounting for approximately 30% of congenital neck disease (1, 16). The most widely accepted theory is that the BCAs result from incomplete obliteration of branchial pouches and clefts during embryogenesis (1, 8). Of these, CSBCAs are the most common, making up 90%–95% of all BCAs (1, 2, 5, 13). As in some previous studies, there was no predilection for location [13 right (52.0%) and 12 left (48.0%)] and sex [12/25 males (48.0%) and 13/25 females (52.0%)] of CSBCAs (6, 10). Conversely, a few researchers considered that the lesions were slightly more common on the right side [81% (54/67); 79% (49/62)] and in males (39/68, 57%) (2, 9). In the present study, 36 cases (69.2%) were male and 16 cases (30.8%) were female. The lesions were on the left side in 15 (28.8%) patients, on the right side in 26 (50.0%) patients, and bilaterally in 11 (21.2%) patients (Table 1). Our results supported that CSBCAs tended to occur on the right side and in males. CSBCAs may present as true fistula, sinus tracts, or complete isolated cysts, commonly as cysts (1, 13). Clinically, the cystic lesions usually present as painless, solitary, slow-growing, and fluctuant masses in the lateral neck (7, 8, 13). However, in certain cases, an acute enlargement in the size of the cysts may occur during from an upper respiratory tract infection; tenderness or pain can also occur if cysts are subsequently infected (3, 17). Seventeen patients (32.7%) in our study had a primary complaint of lateral neck swelling, eight patients with asymptomatic mass and the remaining nine patients with pain. A second BC external sinus/fistula opens externally in the neck between the mid and lower third of the anterior border of SCM noted at birth, and becomes evident in early childhood presenting with recurrent lower neck mucopurulent infections or persistent mucoid discharge from a skin opening (2, 12). In the present study, CSBCAs were more frequent as sinuses/fistulas (35/52, 67.3%), and it was consistent with some other reports (18). The sinus/fistula presented as having an external skin opening at the mid-lower SCM region in newborns or discharge from the skin opening. Prior to surgery, 30 patients (57.7%) had a history of infection, including 9 cysts and 21 fistulas/sinuses, and they were treated by antibiotics (Table 1). It lacks characteristic symptoms and therefore is prone to be missed during diagnosis or leads to clinical misdiagnosis (13, 19). As a result, some patients underwent inappropriate procedures, including sclerotherapy, aspiration, incision, and drainage. These procedures were considered to be temporary and noncurative, which can increase the risk of secondary infection and lead to recurrence of the swelling (20). Nineteen patients in our study had a history of incision and drainage for 1–3 times, 1 had undergone the aspiration procedure and 1 had a history of surgical resection at a local hospital. Fourteen patients (27.0%) had a clinically relevant misdiagnosis preoperatively, including LMs, neck abscess, TGDC, and CPSF. Our results were consistent with the above reports (Table 1).

Preoperative imaging assessments for CSBCAs include US, CT, and magnetic resonance imaging (MRI), which can be helpful in confirming the clinical diagnosis and making operative planning (1, 6, 7, 15). US is usually the initial imaging because it is noninvasive, without sedation, and can differentiate between a solid mass and a cyst. However, US evaluation of extension and adjacent vital structures of CSBCAs is limited (1, 6, 7). Therefore, to determine the localization, extent of the lesion, and relationship with surrounding anatomical structures, CT is most commonly used clinically (6, 7, 13, 15). Moreover, CT is readily available and can be quickly obtained (1). In branchial cleft cysts, CT scans show a well-circumscribed and homogeneously hypodense cystic mass with a uniformly thin, smooth wall (7). While the tract may be visible in branchial cleft sinuses/fistulas (6, 13). In this study, we performed CT scans in all the patients, and CT had a diagnostic accuracy of 86.5% (45/52) for CSBCAs. The size of CSBCAs ranged from 0.4 to 6.1 cm (mean, 2.3 cm ± 1.4 cm). In addition, in some cases who had a history of infection, we found that the cyst wall became thickened and irregular, which was consistent with reports (3, 7). Thirty-seven patients underwent neck US. US showed 22 cases of benign cystic mass, 7 cases of fistula, and only 4 cases were accurately diagnosed, so the diagnostic accuracy (10.8%, 4/37) was very low. According to Bailey classification, CSBCAs may be present from the skin on the lateral neck, posterior to the SMG, between the internal and external carotid arteries, to the palatine tonsil (3, 8, 14). Clinically, Bailey type II is the most common, while type IV is the rarest (1, 14, 21). In our series, CSBCAs were classified as type I in 10 (19.2%), type II in 35 (67.3%), type III in 4 (7.7%), and type IV in 3 (5.8%) patients. Type II was the most common and found in the “classic” location: along the anteromedial border of the SCM, lateral to the carotid sheath, and posterior to the SMG (Figures 2D–F). This was basically the same as the above report (3). As MRI examination takes a long time and costs more (22), it was not performed as a routine preoperatively in our department.

The differential diagnosis of CSBCAs contains TGDC, CFBCAs, CPSF, LMs, and other cystic neck lesions (1, 7, 8, 23). On CT, a TGDC shows a low-density, usually unilocular thin-wall lesion along the course of embryologic thyroid migration, mostly lying in the midline neck and closely related to the hyoid bone. Clinically, TGDC presents as anterior neck mass or painless median swelling that moves on swallowing or protruding the tongue (24). The lesions of head and neck LMs are commonly non-enhancing, poorly circumscribed, and with infiltrative growth along the tissue space, and the fibrous septum are strengthened in strip or grid shape in multilocular cysts (25, 26). The LMs mainly show a smooth surface, soft texture, obvious wave motion, and positive light transmission test. LMs are usually diagnosed within the first 2 years of life, and 50% of LMs are present at birth (26). CFBCAs universally occur in Pochet's triangle area (above the hyoid bone, posterior to the submandibular angle, and below the external auditory canal), particularly in the retroauricular groove or parotid region, which are closely related to parotid gland, facial nerve, and external auditory canal (15, 27). CFBCAs may present as having an external opening in the submandibular or periauricular at birth, while internal opening is found in the external auditory canal (15). For CPSF, the CT generally revealed lesions in the left side and invasion of thyroid tissue, abscess with air, the shallower or disappearing pyriform sinus, tubular structures seen inside the thyroid gland, and gas-containing ducts originating from the piriform fossa (10, 28). The main clinical manifestations of CPSF are recurrent neck abscess and suppurative thyroiditis (10, 28). In this study, 14 children (27.0%) had a clinically relevant misdiagnosis preoperatively, mainly including LMs, neck abscess, TGDC, and CPSF. Therefore, being familiar with the characteristic CT features of CSBCAs will help clinicians to accurately diagnose and formulate surgical strategies.

Complete open surgical excision of the CSBCAs is considered the standard treatment (6, 8, 9). Some authors have suggested not only excision of the fistula/sinus tract but also ipsilateral tonsillectomy, considering the tract terminates at the tonsillar fossa (29). Recent studies have found that recurrence was not related to unilateral tonsillectomy, but high ligation of the fistula was sufficient to avoid recurrence (2, 9, 15, 30). However, recurrence rate may vary from 3% to 22% if there was a history of multiple infections or incomplete excision (2, 8). In this study, open surgery was carried out under general anesthesia during noninfectious stage, *via* lateral neck incision, and 98.1% cases (51/52) of CSBCAs had no ipsilateral tonsillectomy. The remaining one underwent ipsilateral tonsillectomy because the mass had protruded to the oropharynx (Figure 4B). After a median follow-up of 30 months (range 4–90), we found no recurrence or complications after surgery, even in children with a history of infection, aspiration, surgery, or incision and drainage. Our findings support that high ligation is sufficient for complete excision of CSBCAs (Figure 2C). Recently, several attempts, such as endoscopic excision, robot-assisted excision, or chemocauterization of the fistula tract, have been made to avoid a visible scar (12, 31). However, whether these techniques can replace the traditional open surgery remains to be supported by more cases and longer term follow-up (15).

It is crucial for the surgeon to fully comprehend the clinical characteristics and typical CT features of CSBCAs. The limitation of this study was the small sample size, from a single center, and the retrospective review.

## 5. Conclusion

The Bailey type II was the most common among CSBCAs. The characteristic CT features of CSBCAs are well-circumscribed cystic mass in lateral neck with a smooth, thin wall, and the lesions are commonly located at the anteromedial border of the SCM, posterior to the SMG, and lateral to the carotid space. Surgical excision without ipsilateral tonsillectomy, generally, is the treatment of choice without any complications and recurrence.

### 5.1. Future perspectives

CSBCAs are common diseases, but they are often misdiagnosed, receiving inappropriate or even harmful intervention. In follow-up clinical research, we will introduce the methods of artificial intelligence and radiomics to further achieve the accurate diagnosis and treatment of CSBCAs.

## Data Availability

The original contributions presented in the study are included in the article, further inquiries can be directed to the corresponding author.
